# Further Evidence of a Recessive Variant in *COL1A1* as an Underlying Cause of Ehlers–Danlos Syndrome: A Report of a Saudi Founder Mutation

**DOI:** 10.1055/s-0041-1722873

**Published:** 2021-02-01

**Authors:** Ahmad Almatrafi, Jamil A. Hashmi, Fatima Fadhli, Asma Alharbi, Sibtain Afzal, Khushnooda Ramzan, Sulman Basit

**Affiliations:** 1 Department of Biology, College of Science, Taibah University, Almadinah Almunawwarah, Saudi Arabia; 2 Center for Genetics and Inherited Diseases, Taibah University Almadinah Almunawwarah, Medina, Kingdom of Saudi Arabia; 3 Department of Genetics, Madinah Maternity and Children Hospital, Medina, Kingdom of Saudi Arabia; 4 Faculty of Allied and Health Sciences, Imperial College of Business Studies, Lahore, Pakistan; 5 Department of Genetics, King Faisal Specialist Hospital and Research Centre, Riyadh, Kingdom of Saudi Arabia

**Keywords:** COL1A1 homozygous mutation, Ehlers–Danlos syndrome, founder mutation, Saudi family

## Abstract

Ehlers–Danlos syndrome (EDS) is a group of clinically and genetically heterogeneous disorder of soft connective tissues. The hallmark clinical features of the EDS are hyperextensible skin, hypermobile joints, and fragile vessels. It exhibits associated symptoms including contractures of muscles, kyphoscoliosis, spondylodysplasia, dermatosparaxis, periodontitis, and arthrochalasia. The aim of this study is to determine the exact subtype of EDS by molecular genetic testing in a family segregating EDS in an autosomal recessive manner. Herein, we describe a family with two individuals afflicted with EDS. Whole exome sequencing identified a homozygous missense mutation (c.2050G > A; p.Glu684Lys) in the *COL1A1* gene in both affected individuals, although heterozygous variants in the *COL1A1* are known to cause EDS. Recently, only one report showed homozygous variant as an underlying cause of the EDS in two Saudi families. This is the second report of a homozygous variant in the *COL1A1* gene in a family of Saudi origin. Heterozygous carriers of *COL1A1* variant are asymptomatic. Interestingly, the homozygous variant identified previously and the one identified in this study are same (c.2050G > A). The identification of a unique homozygous mutation (c.2050G > A) in three Saudi families argues in favor of a founder effect.

## Introduction

Ehlers–Danlos syndrome (EDS) is an inherited disorder of connective tissues and comprises a wide range of clinical conditions involving skin, joints, and vessels. EDS manifests as joint hypermobility, skin hyperextensibility, and vascular fragility. 1 2 EDS is phenotypically heterogeneous and a variety of other associated clinical features have been reported, including severe periodontitis, multiple congenital contractures, ocular malformations, kyphoscoliosis, and arterial and intestinal ruptures. 3 4 5 6 7

Inheritance pattern in EDS is both an autosomal recessive as well as an autosomal dominant. The disorder is genetically heterogeneous and at least six autosomal dominant and seven autosomal recessive forms of the EDS have been identified and, except in one case, the corresponding genes have been discovered. The autosomal dominant form of the EDS have been shown to result from mutations in the genes *COL1A1* , *COL1A2* , *COL3A1* , *COL5A1* , *COL5A2* , *COL12A1* , *C1R* , and *C1S* . 5 8 9 10 11 12 13 14 Whereas mutations in *B4GALT7, B3GALT6, SLC39A13, ADAMTS2, TNXB, PLOD1, FKBP14 ZNF469, PRDM5 CHST14, DSE,* and *AEBP1* genes have been shown to cause autosomal recessive form of the EDS. 15 16 17 18 19 20 21 22 23 24 Recently, Alazami et al have shown that homozygous mutation in the *COL1A1* gene can also lead to an autosomal recessive form of EDS. 23 Although, mutations in 19 different genes have been identified as an underlying cause of the 12 different EDS types, much remains to be determined clinically and molecularly about EDS phenotype and EDS like spectrum. 25

Here, we presented a five generations consanguineous Saudi family with suspected symptoms of EDS. Considering the huge genetic heterogeneity, whole exome sequencing strategy was undertaken. Genetic data analysis identified a homozygous mutation in the *COL1A1* as an underlying cause of EDS in the family.

## Materials and Methods

### Ethical Approval and Sample Collection/Pedigree Information

**Fig. 1:**
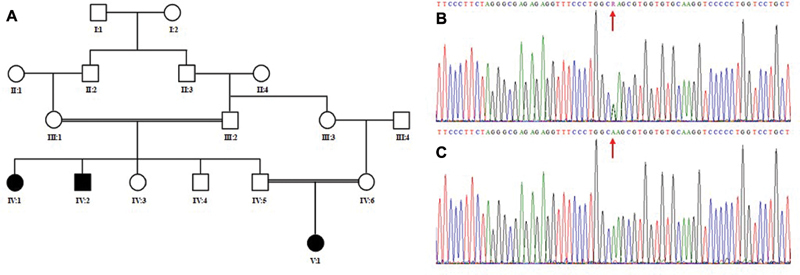
( **A** ) Pedigree of the family investigated in the present study showing autosomal recessive inheritance pattern. Double lines indicate consanguineous marriages. Filled symbols represent affected individuals. ( **B, C** ) Partial sequence of the exon 31 of the *COL1A1* gene obtained by Sanger sequencing. Upper panel ( **B** ) shows chromatogram of the carrier and lower panel ( **C** ) shows chromatogram of the affected individuals of the family. Red arrow indicates the position of the mutation.

Ethical approval to commence the work was obtained from the ethical review committee of Taibah University Almadinah Almunawarah, Kingdom of Saudi Arabia. A 5-generation Saudi consanguineous family with two affected individuals was referred to the Center for Genetics and Inherited Diseases (CGID) from Madinah Maternity and Children Hospital (MMCH) by a pediatric consultant for detailed molecular investigation. Informed written consents were taken from all the members of the family; parents gave consents in case of children. Family history of the disease was enquired from the parents, and a pedigree was drawn based upon the information taken from elders of the family ( Fig. 1A ).

### Clinical Evaluation

A complete clinical checkup of the affected members was conducted at MMCH by a pediatric consultant. Skin and musculoskeletal system was evaluated. A Beighton score was used to assess the joint hypermobility. Full blood count and echocardiography were performed.

### DNA Extraction

Approximately 5 mL peripheral blood sample was collected from affected as well as unaffected members of the family in EDTA blood vacutainers. Genomic DNA from these blood samples were extracted using QIAquick DNA extraction kit. Nanodrop spectrophotometer and gel electrophoresis were used to evaluate the quality and integrity of the extracted DNA, respectively.

### Whole Genome Single Nucleotide Polymorphism (SNP) Genotyping

Whole genome SNP genotyping array was performed using Illumina iScan platform and HumanOmni 2.5M bead chip, genotyping 2.5 million SNPs. Approximately 200 ng genomic DNA of two affected and two unaffected members were taken for genotyping as per protocol described elsewhere. 26 27 Illumina genome studio and homozygosity mapper 28 29 were employed to detect common regions sharing homozygosity amongst the affected members.

### Whole Exome Sequencing (WES)

In an attempt to identify the underlying genetic variant causing EDS, entire coding region (whole exome) was sequenced using Nextera rapid capture exome kit and Illumina NextSeq500 machine. A total of 70 ng of the genomic DNA from both the affected members were used as a starting material, while library preparation and exome enrichment were done using Nextera rapid capture exome kit. Detailed protocol used for WES was the same as described elsewhere. 30

### Candidate Gene Validation by Sanger Sequencing

Genetic analyzer ABI3500 was used to validate the genetic variant discovered by WES. Primer 3 31 software was used to design the primers from the flanking regions of the candidate variants. Detailed protocol that was used for Sanger validation was the same as described elsewhere. 32 33 The sequenced variants were identified using BIOEDIT sequence alignment editor version 6.0.7 (Ibis bioSciences Inc.; CA, USA).

## Results

### Clinical Evaluation Determined the EDS Features

The family was clinically evaluated by a consultant at MMCH. Affected members of the family manifested typical symptoms of the EDS type 1, including skin loosening (hyperextensivity of the skin), tissue fragility, and bruised skin with variable degree of expression amongst the two members. Unaffected members of the family including normal siblings and parents did not show any of these disease symptoms. A Beighton score of 5 was obtained, indicating a generalized joint hypermobility. Full blood count was normal and clotting disorders were excluded.

### Homozygosity Mapping Identified a Loss of Heterozygosity (LOH) Region on Chromosome17q21.33

A common region of homozygosity shared by both the affected members of the family was identified by whole genome SNP genotyping array with the help of 2.5M Illumina iScan platform. Healthy members of the family did not share this homozygosity region. The shard homozygous region is of 4 Mb on chromosome 17q21.33.

### WES Analysis Identified a Homozygous Variant in COL1A1 Gene

An average coverage (covering 214405 exons and splice sites) of 98.3% was obtained from exome sequencing. Different suitable filter options were employed to analyze WES data generated by Illumina NextSeq500 platform. The shared homozygous region identified by genome-wide SNP genotyping array was primarily targeted due to its disease relevance, in order to identify underlying gene of interest. A missense homozygous variant (c.2050G > A) in the exon 31 of the *COL1A1* gene was identified. The gene *COL1A1* is present in the shared homozygous region. Besides targeting this shared homozygous region, different filter options like pathogenicity, allele frequency, genomic position, nonsynonymous variants, protein effect, etc., were used to further filter the entire 90,000 genetic variants. However, we failed to detect any other notable, relevant genetic variants responsible for the EDS phenotype. Therefore, a missense variant (c.2050G > A; p.Glu684Lys) in exon 31 of the *COL1A1* gene was considered as the genetic defect underlying EDS phenotype in the family.

### Sanger Sequencing Validate the Variant and Segregation Analysis

The variant present in the exon 31 of the *COL1A1* gene was validated by Sanger sequencing approach. Data analysis validated the presence of the variant (c.2050G > A) and its complete segregation within the family. Variant (c.2050G > A) was found in homozygous state in the DNA of affected individuals, while unaffected members of the family were either heterozygous or wild type ( Fig. 1B ).

## Discussion

Heterozygous mutations in type 1 collagen gene, *COL1A1* and *COL1A2* , are frequently known to cause osteogenesis imperfecta. 34 35 36 Osteogenesis imperfecta type 1 can be confused with EDS syndrome, however, a detailed physical examination along with combination of clinical features can help distinguish the two. 37

Patients with inherited connective tissue disorders present with diverse clinical features, and differential diagnosis is challenging. 23 In such cases, clinical distinction is not always straightforward for the physician. We presented a Saudi family with two members afflicted with connective tissue disorders. Complete clinical evaluation diagnosed the individuals with a classic type of Ehlers–Danlos syndrome (cEDS). Patients demonstrated skin fragility as a bruising and poor wound healing. Surgical scars were evident on legs. Interviewing elders revealed presence of individuals with similar clinical features in another loop of the family. Family pedigree was found consistent with an autosomal recessive inheritance pattern.

cEDS with typical disease symptoms included exceptional high skin extensibility and fragility, hypermobility of joints, atrophic scared and bruised skin. Molecular investigations led to the identification of a homozygous missense variant (c.2050G > A) in the *COL1A1* gene. This is the second report of a homozygous variant in the *COL1A1* in EDS patients. The clinical features including respiratory distress at birth, severe hypotonia with muscle wasting, head lag, and absent reflexes, reported earlier in a family with same homozygous mutation (c.2050G > A) in *COL1A1* have not been observed in our cases. This report provides further evidence that COL1A1 homozygous variants may cause EDS like phenotype, and the variant identified in this study is a founder in Saudi population.
